# Influence of Oil Status on Membrane-Based Gas–Oil Separation in DGA

**DOI:** 10.3390/s22103629

**Published:** 2022-05-10

**Authors:** Tunan Chen, Kang Li, Zhenghai Liao, Xiongjie Xie, Guoqiang Zhang

**Affiliations:** 1 Institute of Electrical Engineering, Chinese Academy of Sciences, Beijing 100190, China; tnchen@mail.iee.ac.cn (T.C.); likang07@mail.iee.ac.cn (K.L.); 2 University of Chinese Academy of Sciences, Beijing 100049, China; 3 State Key Laboratory of Power Grid Environmental Protection, China Electric Power Research Institute, Wuhan 430074, China; liaozhenghai@epri.sgcc.com.cn (Z.L.); xiexiongjie@epri.sgcc.com.cn (X.X.)

**Keywords:** gas–oil separation, membrane, photoacoustic spectroscopy

## Abstract

Gas–oil separation by membrane stands for a promising technique in dissolved gas analysis (DGA). Since the accuracy of DGA relies on the results of gas–oil separation to a great extent, it is necessary to study the influence factor of membrane for better performance. Although plentiful studies have been conducted aiming at membrane modification to obtain better separation performance, it cannot be ignored that the conditions of oil also affect the performance of membrane much. In this work, a photoacoustic spectroscopy-based sensor for DGA, which employed membrane for gas–oil separation, was established first. By detecting the photoacoustic signal, the performance of membrane could be evaluated. Furthermore, the influences of feed velocity and pressure have on the performance of membrane were analyzed. Both simulation and experiment were employed in this work to evaluate the influences by collecting the equilibrium time of membrane under different conditions. As a result, the simulation and experiment agreed with each other well. Moreover, it was reasonable to draw the conclusion that the equilibrium time was evidently reduced with the raise of feed velocity but remained with a minimum change when pressure changed. The conclusion may serve as a reference for the application of membrane in optical sensor and DGA.

## 1. Introduction

Oil-immersed electrical equipment plays a critical role in electrical energy transmission and distribution. The safe and stable operation of such equipment has long been the center of electrical industry. When insulation faults occur, a series of decomposition product will be generated. The category and concentration of those decomposition products strongly correlate with the insulation status of transformer. Based on current studies, acetylene (C_2_H_2_) is generally considered as the most significant decomposition of insulation oil which can stand for the severity of discharge fault in oil-insulated equipment [1,2]. To monitor the concentration of dissolved C_2_H_2_, dissolved gas analysis (DGA) is often considered as the most efficient and convenient way [3].

In order to diagnose potential insulation fault at early stage, high sensitivity C_2_H_2_ detection method is required. Compared with conventional gas chromatographic (GC)-based method, photoacoustic spectroscopy (PAS)-based method is with higher accuracy and shorter response time [4,5]. Hence, PAS is often employed for highly sensitive DGA and has become more and more approved in the electrical industry [6]. Nowadays, several kinds of PAS-based commercial DGA equipment have launched the market and they are now gradually replacing conventional GC-based equipment [7].

On the other hand, dissolved gases need to be extracted first from oil for subsequent analysis in DGA. Under this circumstance, gas–oil separation method affects the results of DGA drastically. Based on the document published by American Society for Testing and Material (ASTM), extraction methods for dissolved gas include vacuum extraction, stripping and headspace [8]. Aforementioned methods usually require complex mechanical devices and considerate maintenance cost. However, for the electrical equipment with limited space and special location, such as bushing, regular floor type DGA equipment with large size is not applicative. Consequently, DGA equipment with low maintenance frequency and small volume is required. As a novel and promising method, membrane-based gas–oil separation method possesses some merits such as energy saving, cost competitiveness and small volume, therefore, membrane technique stands for a kind of promising perspective for DGA [9]. The application of membrane in DGA is beneficial to miniaturizing the size of DGA equipment. Such membrane has been used in small-size commercial DGA equipment [10].

Compared with conventional gas extraction method such as vacuum extraction, the separation efficiency of membrane is limited [11]. Plentiful studies have been conducted to obtain better performance of membrane [12,13]. According to existed researches, the performance of membrane is primarily decided by the status of membrane itself [14]. However, in practical scenarios, the separation processes are also affected by the surrounding conditions such as pressure and feed velocity [15,16]. Under different conditions, the performance of membrane varies. Thus, it is necessary to study the influences of the conditions of oil on the performance of membrane unit since the results of gas–oil separation is fundamental to the accuracy of DGA.

To develop a DGA equipment with both high accuracy and small size, a PAS-based trace C_2_H_2_ detection system with membrane unit was established in this work. By setting different conditions, the gas–oil separation process could be observed by collecting photoacoustic signal, therefore, the performance of membrane could be evaluated this way. This work evaluated the performances of membrane under different feed velocity and pressure from both simulation and experiment perspectives. Based on the results of this work, the influences of conditions of oil have on the performance in practical scenarios can be learnt, which may serve as a reference for membrane application in DGA.

## 2. Theoretical Fundamentals

### 2.1. Photoacoustic Spectroscopy

PAS detection method is based on gas photoacoustic effect, which is a process from light to sound. In photoacoustic cell, acoustic waves are generated because of periodic transit from gas molecules excited by periodic light. Then, the acoustic waves are collected by microphone and converted to electrical signals. Based on current research, the relationship between converted electrical signals and concentration of investigated gas can be expressed as Equation (1) [17].

(1) $$S_{\mathrm{PAS}}=s_{m}P_{input}F_{cell}\alpha c$$

where, *S*_PAS_ represents the electrical signal (V), *s_m_* represents the sensitivity of the microphone (V/Pa), *P_input_* represents the power of the light source (W), *F_cell_* represents the cell-specific constant (Pa·cm/W), *α* represents the absorption coefficient of investigated gas at the nominal wavelength of the light source (cm^−1^), *c* represents the concentration of investigated gas (ppm).

### 2.2. Non-Porous Membrane

Non-porous membrane is the most recognized category of membrane for gas–oil separation. According to existed studies, a prevailing model to describe non-porous membrane was solution-diffusion model [18,19]. In this model, with the assumption of total pressure being 1 atm, the concentration in the downstream side, which was the gas room in this work, could be calculated as Equation (2).

(2) $$c_{g}=\left(9.87kc_{0}-c_{g}{}_{0}\right)\left[1-\exp\left(-\frac{10^{5}HA}{Vd}t\right)\right]+c_{g}{}_{0}$$

where *c_g_* represents the concentration of gas (ppm), which is C_2_H_2_ in this work, in the gas room after time *t*, *k* represents the equilibrium constant, *c*_0_ represents the gas concentration in the oil (ppm), *c_g_*_0_ represents the initial gas concentration in the gas room (ppm), *H* represents the permeability of gas in the membrane (cm^2^/(s·Pa)), *A* represents effective contacting area between membrane and oil (cm^2^), *t* represents time (s), *d* represents the thickness of membrane (cm), *V* represents the volume of gas room (mL).

Usually, the gas room was filled with ambient gas in the first place. Therefore, Equation (2) could be rewritten as Equation (3).

(3) $$c_{g}=9.87kc_{0}\left[1-\exp\left(-\frac{10^{5}HA}{Vd}t\right)\right]$$

Plus, equilibrium constant is related to the category of gas instead of membrane only. In practical scenario, equilibrium can be seen as achieved when the concentration of investigated gas reaches 90% of theoretical maximum. Therefore, the equilibrium time can be calculated as Equation (4) [20,21].

(4) $$t=\frac{2.3Vd}{10^{5}HA}$$

In this work, the equilibrium times were collected in this way.

### 2.3. Concentration Polarization

For a dissolved gas brought to a membrane surface, there is an accumulation of solute gas in the boundary layer adjacent to the membrane. Such phenomenon, which is referred to as concentration polarization, severely impairs membrane separation processes [22,23]. Thus, this reduces the performance of membrane and raises the costs of capital and operation. The concentration polarization of the investigated gas can be described as Equations (5)–(7) [24].

(5) $$\mathrm{CP}=1-\frac{JF\left(1-x\right)\left(1-\beta \right)}{k+JF\left[1-x\left(1-\beta \right)\right]}$$

(6) $$F=P_{0}\frac{T}{T_{0}}\left(1-\frac{P_{p}}{P_{f}}\right)$$

(7) $$\beta =\frac{1}{\alpha_{0}}$$

where, *J* represents the permeation rate of the gas, *x* represents the molar fraction of the gas in the bulk feed, *k* represents the mass transfer coefficient which is proportional to the velocity of the feed gas, *P*_0_ represents standard pressure, *T* represents the temperature on the feed side, *T*_0_ represents standard temperature, *P_p_* represents the pressure on the permeate side, *P_f_* represents the pressure on the feed side, *α*_0_ represents the separation factor of the gas without concentration polarization.

## 3. Simulation Modeling and Experiment Setups

Based on the theory aforementioned, this paper employed both simulation and experiment and testified the influence of the conditions of fluid to the performance of membrane. Fundamental preparations of simulation and experiment were introduced successively as follows.

### 3.1. Simulation Modeling

In this work, COMSOL Multiphysics was employed in this work to simulate the dissolution process. Therefore, the qualitative impact of different feed velocity has on the permeability of membrane could be directly demonstrated.

**Figure 1:**
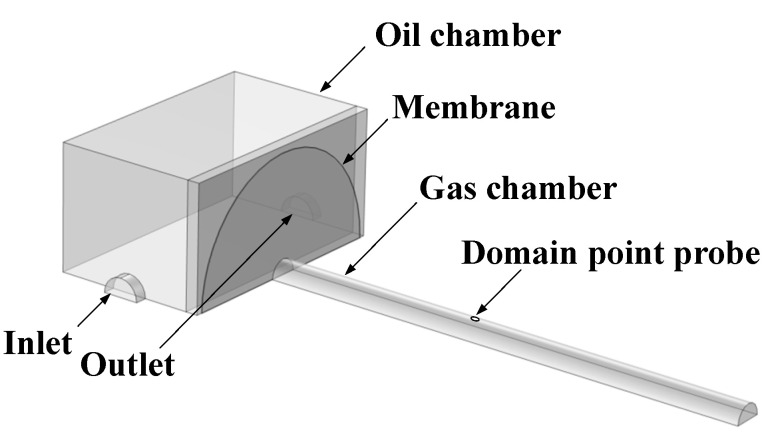
Basic structure of simulation modeling.

Considering the symmetry of the structure, a symmetrical model that demonstrated upper half of integration photoacoustic cell was established in COMSOL Multiphysics, which is shown in Figure 1.

From Figure 1, inlet and outlet were the entrance and exit of oil, with a domain point probe placed in the top center of gas room to detect the concentration of gas.

### 3.2. Experimental Setups

**Figure 2:**
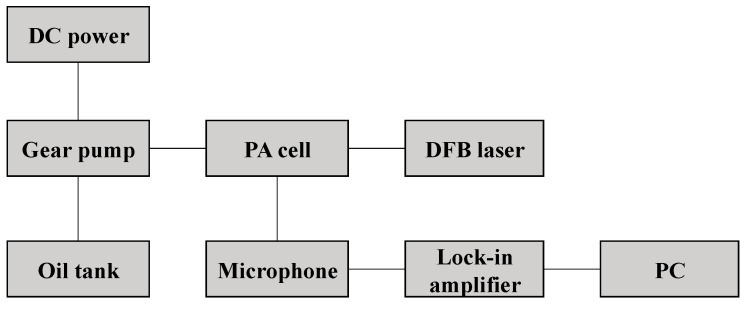
Topological structure of the detection system.

In this work, the concentration of investigated gas was reflected by the value of photoacoustic signal. To monitor the change of the concentration of investigated gas, a detection system based on PAS was established in this work, whose topological structure is shown in Figure 2.

**Figure 3:**
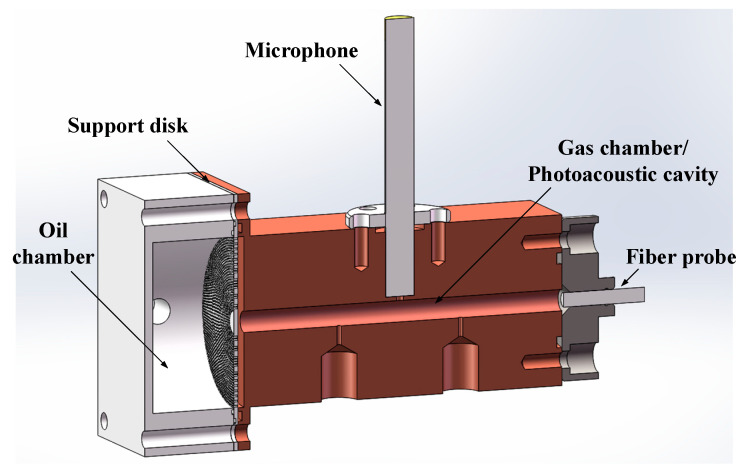
Structure of integration photoacoustic cell.

In detail, an integration photoacoustic cell was designed for gas–oil separation and detection. The structure is shown in Figure 3.

From Figure 3, flat membrane unit was employed in this paper. With the thickness of 12.5 μm, the membrane in this work was made of fluorinated ethylene propylene (FEP). The oil contacted constantly with the membrane in the form of cross fluent. After dissolved gas was separated from oil by membrane, it diffused into the gas room for detection. The integration design of the photoacoustic cell was to reduce the volume of gas room and shorten balance time accordingly. Plus, the speed of gear pump was proportional to its voltage. Therefore, the feed velocity of oil could be adjusted by controlling the output of DC power.

## 4. Results and Discussions

In this section, the influences of the conditions of oil feed velocity on the performance of membrane were discussed by analyzing the simulation and experiment results. The influences of feed velocity and pressure were studied successively.

### 4.1. Simulation Results and Analysis

In this work, the influence of feed velocity on membrane performance was studied from both simulation and experiment side, which would be introduced successively.

**Figure 4:**
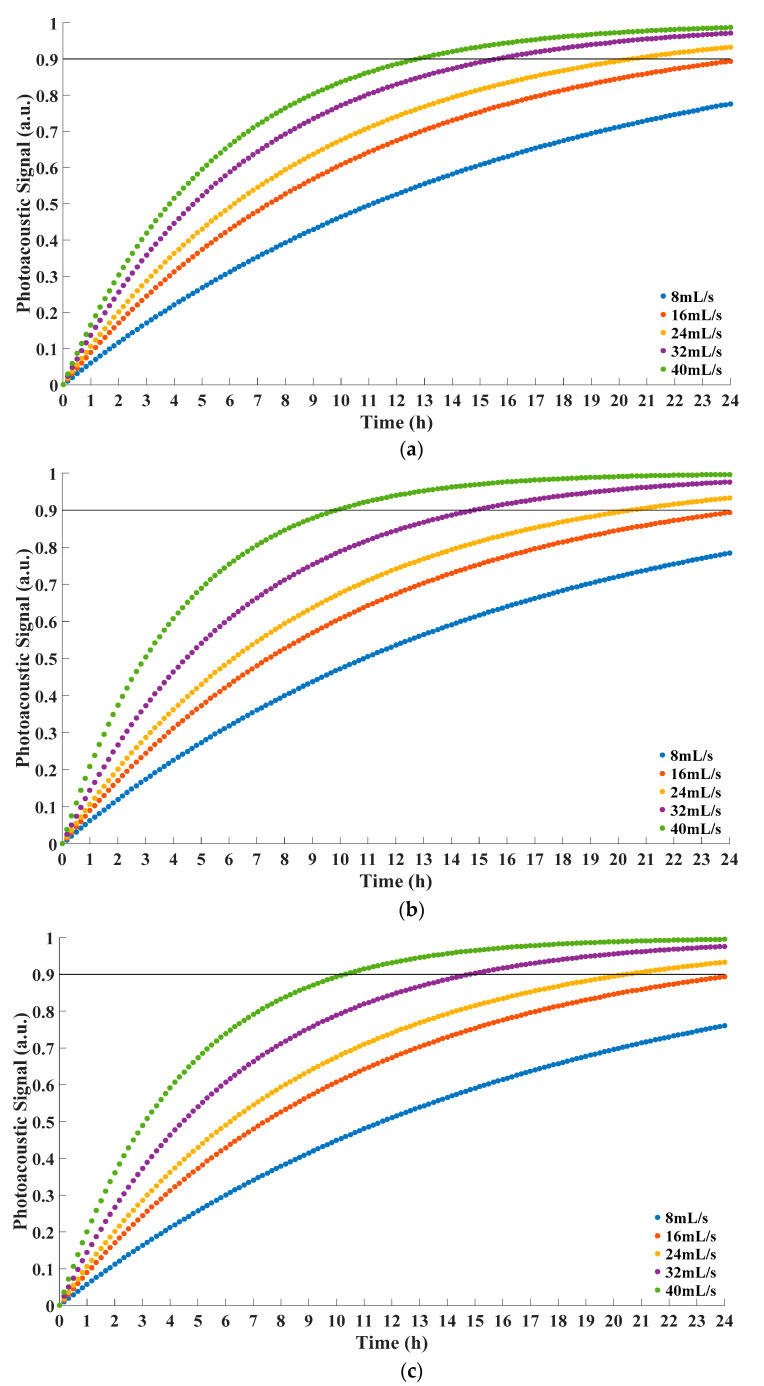
Concentration at domain point probe under different feed velocity. (**a**) Pressure 1.0 atm; (**b**) Pressure 1.5 atm; (**c**) Pressure 2.0 atm.

Firstly, in COMSOL Multiphysics, we set the pressure of oil fluid as 1.0 atm. Then, we set the feed velocity at the inlet in the simulation model as 8 mL/s, 16 mL/s, 24 mL/s, 32 mL/s, 40 mL/s, successively. Next, we conducted time-dependent study at each condition. Finally, we repeated aforementioned procedure with the pressure of 1.5 atm and 2.0 atm. Simulation results of the concentration at domain point probe are shown in Figure 4.

According to Equation (1), the concentration of investigated gas on the permeate side was proportional to the value of photoacoustic signal. Therefore, the separation process of membrane could be indicated by the variation of the gas concentration in the gas room. On the other hand, the relationship between time and photoacoustic signal was consistent with Equation (3) in the form and *R*^2^ > 0.999 testified the correctness of applying solution-diffusion model.

From Figure 4, it could be seen that the equilibrium time decreased with the increasement of the feed velocity. The increase of feed velocity reduced the level of concentration polarization and contributed to the permeation of gas in the membrane. Therefore, the equilibrium time was shortened as well. For better demonstration, the equilibrium times are listed as Table 1.

From Table 1, it could be seen that the equilibrium time drastically decreased when the feed velocity increased, which indicated that the flux increased accordingly. Based on Equation (5), the concentration polarization decreased with the increase of the feed velocity. Consequently, the performance of membrane was improved. So, it could be concluded that the feed velocity of oil should be as large as possible to obtain shorter equilibrium time.

Moreover, with the same feed velocity, the variation of pressure affected the equilibrium time little. Considering the actual fluctuation of pressure in the equipment was usually smaller than the pressure difference in our simulation, it could be concluded that the equilibrium time was barely impacted by the pressure of oil. This indicated that the performance of membrane was rather stable if the pressure varies [15].

### 4.2. Experimental Results and Analysis

Based on the topological structure aforementioned in Figure 2, a detection system for trace C_2_H_2_ was established in this work to monitor the performance of membrane. Then, we set the pressure of oil as 1.0 atm, 1.5 atm, 2.0 atm, respectively. Under same pressure condition, we set the speed of gear pump at 800 rpm, 1600 rpm, 2400 rpm, 3200 rpm, 4000 rpm. Corresponding feed velocity of oil was 8 mL/s, 16 mL/s, 24 mL/s, 32 mL/s, 40 mL/s, respectively. We successively detected the concentration of separated C_2_H_2_ every half hour. After the scatter data were collected, corresponding fitting functions were calculated successively. In this work, BoxLucas model was for fitting. The model can be expressed as Equation (8).

(8) $$y=a\left(1-e^{-bx}\right)$$

where, *y* represents photoacoustic signal, *x* represents time, *a* and *b* are fitting parameters. It could be seen that the equilibrium time to reach 90% of maximum was only related to the value of *b*. In order to obtain direct comparison of equilibrium time under different conditions, experiment data were normalized and *a* was set as 1 in this work.

**Figure 5:**
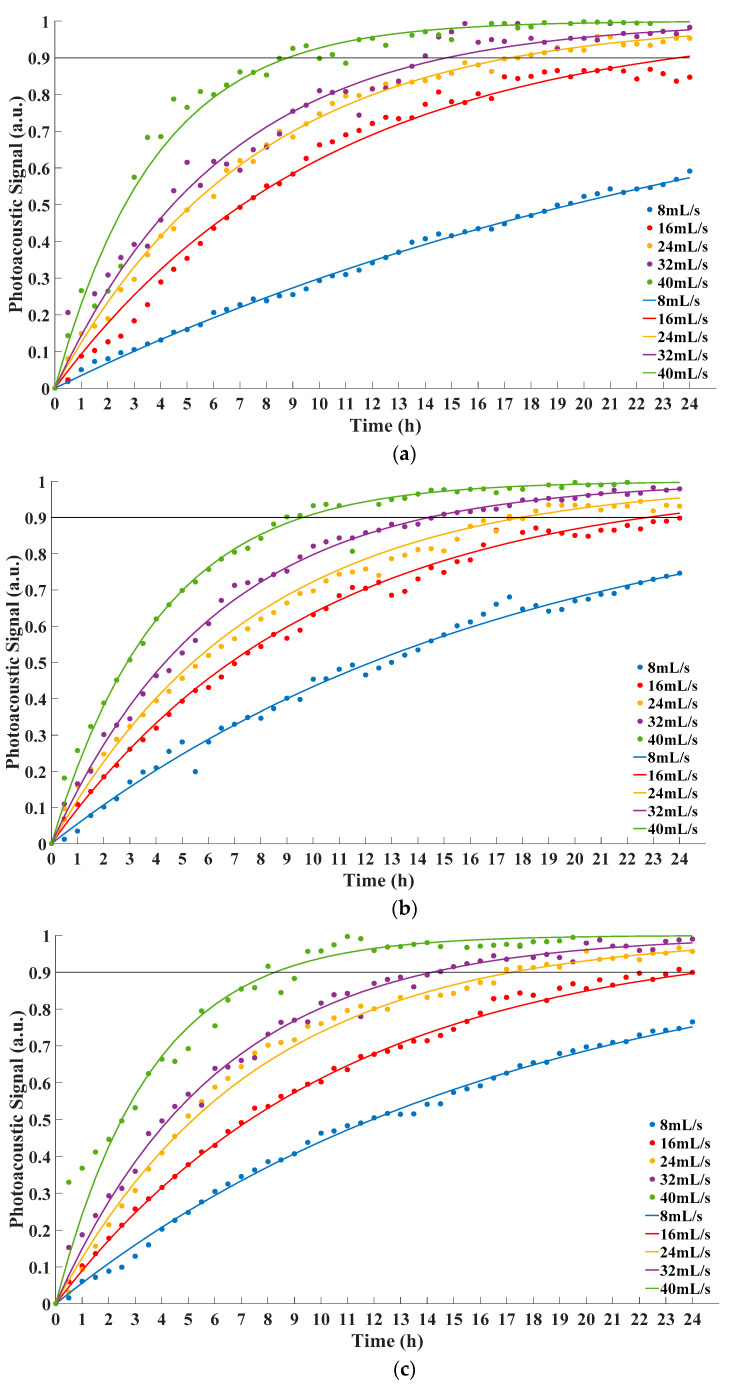
Concentration at gas room under different feed velocity. (**a**) Pressure 1.0 atm; (**b**) Pressure 1.5 atm; (**c**) Pressure 2.0 atm.

The data and corresponding fitting functions were as Figure 5.

The fitting parameters of each curve were listed as Table 2.

From Figure 5 and Table 2, it could be concluded that fitting functions were consistent with Equation (3) in the form. *R*^2^ > 0.95 verified the rationality of the fitting functions. The equilibrium times under different conditions were calculated on the basis of corresponding fitting functions. The equilibrium times in the experiment are listed in Table 3.

From Figure 5 and Table 3, with the increase of the feed velocity, photoacoustic signal increased faster accordingly. Therefore, the time to reach equilibrium decreased. This indicated that the performance of membrane increased with the feed velocity, which agreed with Equation (5). Additionally, the equilibrium time was hardly affected by the pressure of oil on the feed side. The results of experiment demonstrated that it could match simulation results well. Moreover, the experience of the research could also be beneficial to the application in actual scenario. The feed velocity contributed to shortening equilibrium time, but excessive feed velocity would also cause overheating problem because of the friction between oil and boundary. Furthermore, overheating oil was one of the main reasons to generate bubbles [25]. Bubbles could impair the insulation capability of oil and cause partial discharge [26]. Therefore, the feed velocity of oil should be as large as possible within a reasonable range. Although no bubble was generated, the feed velocity in this work was large enough to cause obvious temperature rise. Extra cooling measure was used to keep the temperature of integration photoacoustic cell steady. In a practical scenario, this could be a reference for the equipment to maintain stable performance.

## 5. Conclusions

In this work, the influences of the conditions of oil on the performance of membrane were studied. To be more specific, the performance of membrane under different feed velocity and pressure of oil was analyzed. First of all, relevant theories were introduced to analyze potential influences the conditions of oil had on the performance of membrane.

Next, simulation model and experiment were established to investigate the influences in both ways. Then, simulation and experiment were conducted to study the relationship between equilibrium time and feed velocity and pressure. The results indicated the performance of membrane could be improved by adjusting the conditions of oil to obtain better results of DGA. Finally, the analysis results were further applied to investigate how different conditions were going to influence the membrane performance in practical scenarios. It should be noted that the simulation model represents an ideal situation without any interference from outside. Therefore, compared with simulation, the experiment can simulate the actual scenario better since the experiment equipment was the prototype of the device applied in actual scenario. Still, the simulation results could serve as a guide for an experiment to analyze the performance of membrane under different conditions.

With the prevailing application of novel membrane materials, membrane-based DGA equipment with PAS sensor has become more and more approved in the market. The factors that may affect the outcome of DGA should be attached with much importance. Under this circumstance, the results of this work may serve as a guideline for the application of membrane in DGA, especially the scenarios with limited space where the membrane unit is more suitable than conventional gas extraction methods.

## Figures and Tables

**Table 1:** Equilibrium time under different conditions in simulation.

	Pressure	1.0 atm	1.5 atm	2.0 atm
Feed Velocity				
8 mL/s		36.94 h	36.07 h	38.69 h
16 mL/s		24.62 h	24.64 h	24.66 h
24 mL/s		20.34 h	20.45 h	20.47 h
32 mL/s		15.59 h	14.81 h	14.80 h
40 mL/s		12.74 h	9.85 h	10.29 h

**Table 2:** Fitting parameters of each curve.

	Pressure	1.0 atm	1.5 atm	2.0 atm
Feed Velocity				
8 mL/s	*b*	0.01776	0.02837	0.02903
	*R* ^2^	0.99348	0.99183	0.99223
16 mL/s	*b*	0.04887	0.04942	0.04730
	*R* ^2^	0.98667	0.99368	0.98613
24 mL/s	*b*	0.06685	0.06126	0.06691
	*R* ^2^	0.99627	0.99497	0.95639
32 mL/s	*b*	0.07776	0.07990	0.08116
	*R* ^2^	0.95587	0.99662	0.99218
40 mL/s	*b*	0.13081	0.11994	0.13926
	*R* ^2^	0.96630	0.98908	0.96542

**Table 3:** Equilibrium time under different conditions in experiment.

	Pressure	1.0 atm	1.5 atm	2.0 atm
Feed Velocity				
8 mL/s		64.83 h	40.58 h	39.66 h
16 mL/s		23.56 h	22.74 h	24.34 h
24 mL/s		17.22 h	17.94 h	17.21 h
32 mL/s		14.81 h	14.41 h	14.19 h
40 mL/s		8.80 h	9.60 h	8.27 h

## Data Availability

Data sharing not applicable.
